# *Nigella sativa* seed decreases endothelial dysfunction in streptozotocin-induced diabetic rat aorta

**Published:** 2016

**Authors:** Abbasali Abbasnezhad, Saeed Niazmand, Maryam Mahmoudabady, Mohammad Soukhtanloo, Seyed Abdolrahim Rezaee, Seyed Mojtaba Mousavi

**Affiliations:** 1 *Department of Physiology, School of Medicine, Mashhad University of Medical Sciences, Mashhad, Iran *; 2 *Cardiovascular Research Center, Mashhad University of Medical Sciences, Mashhad, Iran*; 3 *Neurogenic Inflammation Research Center, Mashhad University of Medical Sciences, Mashhad, Iran*; 4 *Department of Biochemistry, School of Medicine, Mashhad University of Medical Sciences, Mashhad, Iran*; 5 *Department of Immunology, School of Medicine, Mashhad University of Medical Sciences, Mashhad, Iran*

**Keywords:** *Diabetes mellitus*, *Nigella sativa*, *Endothelial dysfunction*, *Isolated aorta*, *Rat*

## Abstract

**Objective:**

Diabetes is an important risk factor for cardiovascular events. The great percent of morbidity in patients with diabetes is due to endothelial dysfunction. The present study investigated the effects of hydroalcholic extract of *Nigella sativa* (*N. sativa*) on contractile and dilatation response of isolated aorta in streptozotocin (STZ)-induced diabetic rat.

**Materials and Methods:**

Rats were divided into six experimental groups (control, untreated STZ-diabetic, and *N. sativa* hydroalcholic extract or metformin-treated diabetic rats). Treated rats received *N. sativa* extract (100, 200, and 400 mg/kg) or metformin (300 mg/kg) by gavage, daily for 6 weeks.

Isolated rat thoracic rings were mounted in an organ bath system then contractile and dilatation responses induced by phenylephrine (PE), acetylcholine (ACh), potassium chloride (KCl), and sodium nitroprusside (SNP) were evaluated in different situations.

**Results:**

The lower concentrations of *N. sativa* seed extract (DE 100 and DE 200) and metformin significantly reduced the contractile responses to higher concentrations of PE (10^-6 ^- 10^-5 ^M) compared to diabetic group (p<0.05 to p<0.01).

The relaxation response to Ach 10^-8^ M, was increased in DE 200 and metformin groups compared to diabetic group (p<0.05). The relaxation responses to Ach 10^-7^** - **10^-5^ M were significantly higher in all treated groups compared to diabetic group (p<0.05 to p<0.001).

**Conclusion:**

Chronic administration of *N. sativa* seed extract has a significant hypoglycemic effect and improves aortic reactivity to vasoconstrictor and vasodilator agents in STZ-induced diabetic rats.

## Introduction

Diabetes mellitus (DM) and its associated complications are major health problems in the developed world. DM is associated with an increased risk of cardiovascular disease (CVD) even in the presence of intensive glycemic control. Indeed, 75% of diabetic patients will die of CVD (Xu and Zou, 2009 ▶). Diabetic patients have an increased risk for the three major types of macrovascular diseases such as peripheral vascular disease, coronary heart disease, and stroke (Zou et al., 2004 ▶). Endothelial cells potentially regulate basal vascular tone and reactivity in physiological and pathological conditions. Endothelial dysfunction is the main cause of death and disability in diabetic patients (Capellini et al., 2010 ▶). Nitric oxide (NO) is one of the most important factors released by endothelium. Diabetes-induced endothelial dysfunction is characterized by reduced bioavailability of NO in the vessel wall. NO, an important regulator of vascular tone, is produced by the activity of endothelial NO synthase (eNOS) (El-Remessy et al., 2010 ▶) and impaired endothelium-dependent vasodilatation has been well indicated in DM (De Vriese et al., 2000 ▶). 

N. sativa *which* is commonly known as black seed is a plant from the Ranunculaceae family (Ahmad et al., 2013 ▶). N. sativa extact contains 36–38% fixed oils, 0.4–2.5% essential oil, proteins, and alkaloidsand saponins. The fixed oil is composed mainly of fatty acids such as linoleic (C18:2), palmitic (C16:0), oleic (C18:1), and stearic (C18:0) acids (Nergiz and Otles, 1993 ▶). Thymoquinone (TQ) is the most pharmacologically active ingredient found abundantly (30-48%) in the N. sativa, together with its derivatives such as dithymoquinone, thymohydroquinone, and thymol (Ghosheh et al., 1999 ▶).

Several investigations have been shown that N. sativa has a hypoglycemic and antidiabetic effect (Kaleem et al., 2006 ▶). The Neuroprotective effects of N. sativa *and its main constituent have been reported* (Khazdair, 2015 ▶). On the other hand cardiovascular effects of N. sativa *and its main constituent, thymoquinone, such as vasorelaxant (*Niazmand et al., 2014 ▶*),* antihypertensive (Dehkordi and Kamkhah, 2008 ▶; Leong et al., 2013 ▶), hypotensive (Fallah Huseini et al., 2013 ▶), antihyperlipidemic (Ahmad and Beg, 2013), and ameliorative effect of endothelial dysfunction (El-Saleh et al., 2004 ▶) were shown. *N. sativa* can reduce the blood glucose levels by stimulated insulin secretion, increased sensitivity to insulin, inhibition of glucose absorption, reduction of advanced glycation end-products (AGE) accumulation, activation of the AMP-activated protein kinase (AMPK) pathway, and increased expression of muscle glucose transporter 4 (Glut4) (Abbasnezhad et al., 2015 in press). 

The present study was designed to investigate the effects of hydroalcoholic extract of N. sativa seed on aortic reactivity to vasodilator and vasoconstrictor agents in STZ-induced diabetic rats.

## Materials and Methods

**Plant material and preparation of the extract**

*N. sativa* seeds were purchased from local herbal shop in Mashhad, Khorasan province, Iran and identified by botanists in the herbarium of Ferdowsi University of Mashhad (voucher No. 176-2013-9). The seeds were powdered and soaked in 2 L of a hydroalcoholic solution (50% ethanol, 50% water) for 48 h at room temperature. The extraction solution was subsequently filtered and subjected to evaporation under vacuum at 40 °C until the solvent was evaporated. The dried extract was dissolved in the distilled water to obtain the doses of 100, 200, and 400 mg/kg.

**Chemicals and drugs**

All chemicals were of analytical grade (Merck). PE, ACh, KCl, SNP, and STZ were obtained from Sigma (Germany). Moreover, where needed for all drugs, the Krebs solution was used as the solvent. Plasma glucose was measured using Easygluco glucometer (Korea). Serum cholesterol, triglyceride, LDL, and HDL concentrations were determined using Pars Azmoon kits (Tehran, Iran). 

**Animals and induction of diabetes**

Male Wistar rats (250–280 g, 10 weeks old) were housed on a 12 hr light-dark cycle, under constant temperature (22±1 ^o^C) and were allowed free access to standard laboratory diet and drinking water. All experiments were performed under license from the Animal Experimentation Ethics Committee of Mashhad University of Medical Sciences (MUMS).

Diabetes was induced by a single intraperitoneal (IP) injection of STZ (60 mg/kg). Three days after the STZ injection, we confirmed the development of diabetes by measuring fasting blood glucose levels in blood samples taken from tail vein. Rats with blood glucose level≥250 mg/dl were considered to be diabetic. 

**Experimental design**

Rats were randomly assigned to six groups (n = 10 in each group): control (C), diabetic (D), diabetic-metformin (DM), diabetic-extract (DE). Normal saline was administered orally by gavage to the C and D groups, metformin (300 mg/kg) to the DM group, and *N. sativa* seed extract (100, 200, and 400 mg/kg) to the DE groups (DE 100, DE 200, and DE 400) daily by gavage for 6 weeks.

**Preparation of rat aortas**

At the end of the treatment period, all rats were euthanized by decapitation with guillotine. The descending thoracic aorta was rapidly dissected out and immersed in 95% O_2_/5% CO_2_-gassed (carbogen) ice-cold Krebs solution (pH 7.4) with the following composition (mM): NaCl (118.0), KCl (4.7), CaCl_2_ (2.5), KH_2_PO_4_ (1.2), MgSO_4_ (1.6), glucose (11.1), and NaHCO_3 _(25.0). The aorta was removed free of connective tissue and fat and then cut into ring segments of 2–3 mm in length, and care was taken to avoid any damage to the endothelium. The aortic rings were individually suspended on stainless steel rods in 10 ml organ bath containing Krebs solution gassed with carbogen at 37 ^o^C. After a resting tension of 2 g, the vessel segments were allowed to stabilize for 1 h with the bath fluid being changed every 15 min to prevent metabolite interference. Changes in tension were recorded by isometric transducers connected to a data acquisition system (AD instrument, Australia). 

**Experimental procedure**

*Plasma glucose, cholesterol, triglyceride concentrations, and body weight measurements*

Serum fasting blood glucose, cholesterol, and triglyceride concentrations as well as body weight were measured in three different periods of the experiment: before STZ injection, 3 and 24 days after STZ injection (when the diabetes was confirmed), and 6 weeks after STZ injection (day 45).

*Effect of PE and KCl* *on aortic contractile response *

To test the contractile responses of aortic rings, cumulative concentrations of PE (10^-9^ - 10^-^^5^ M) or KCL (20 - 60 mM) were added to the organ bath and the contraction responses were recorded. 

*Effect of Ach and SNP* *on aortic rings dilation response*

To evaluate the vasorelaxant responses of aortic rings, after induction of contraction by PE (10^-6 ^M), cumulative concentrations of Ach (10^-8^ - 10^-5^ M) or SNP (10^-9^ - 10^-6^ M) were added to the organ bath and the relaxation responses were recorded. 

**Data analysis**

Results are expressed as mean±SEM. Statistical analyses were made using one-way ANOVA followed by the Tukey’s post hoc test. Statistical significance was defined as p<0.05.

## Results

**Body weight and plasma glucose, cholesterol, and triglyceride in STZ-induced diabetic and control rats**

The results showed significant weight loss at days 24 and 45 in the diabetic group compared to control group (p<0.001) but in the metformin group, no significant change was observed compared to diabetic group. The weight average at days 24 and 45 in group of DE 400 was less than group of DE 200 (Table 1). Serum fasting blood glucose levels at days 3, 24, and 45 in the untreated diabetic group were significantly different from control group (p<0.001). In the metformin group, glucose level in these periods was not different compared to diabetic group. Our results showed that serum glucose levels in group DE 200 at days 24 and 45 were significantly lower than diabetic group (p<0.05) (Table 1).

**Table 1:** Effect of* N. sativa *seed extract on average weight (g) and serum glucose levels (mg/dl) in streptozotocin-induced diabetic rats

**Group**		**Weight**			**Glucose**				
	**Day 0**	**Day 24**	**Day 45**	**Day 0**	**Day 3**		**Day 24**		**Day 45**
**C**	286.37±428	301.62±4.74	315.12±3.98	86.10±2.62	86.10±2.62		87.38±4.31		84.48±3.96
**D**	278.95±5.7	243.48±6.93	231.43±8.11	82±2.11	335.2±17.54		293.39±7.82		300.54±10.40
**DM**	280.16±5.51	253±4.56	235.5±5.88	98.46±1.67	302.43±10.32		278.99±26.11		267.60±21.31
**DE 100**	273.55±3.95	237.66±7.41	227.22±5.87	97.81±3.91	307.11±13.32		248.56±13.15		238.92±28.59
**DE 200**	292.45±4.80	258.54±9.77	244.81±10.48	91.91±5.89	347.33±8.69		190.99±21.28 *#		168.75±24.32
**DE 400**	282.57±7.75	230.28±13.41	217.85±11.55	91.65±4.05	334.24±5.30		258.74±28.72		227.47±27.40

Abbreviations: C: control, D: diabetic, DM: diabetic + metformin, DE 100: diabetic + *N. sativa *extract of 100 mg/kg, DE 200: diabetic + *N. sativa *extract of 200 mg/kg, DE 400: diabetic + *N. sativa *extract of 400 mg/kg. Values are presented as means±SEM (n = 8).

∗ p<0.05,

** p<0.01 and

*** p<0.001 compared to control group.

# p<0.01,

## p<0.001 and

### p<0.001 compared to diabetic group.

Serum cholesterol levels at days 24 and 45 in the untreated diabetic group were significantly higher than control group (p<0.001). Moreover, cholesterol levels showed significant decrease in all other treated groups compared to untreated diabetic group (p<0.05 to p<0.001) (Table 2). The results showed significant increase of serum triglyceride levels of diabetic groups compared to the control group at days 24 and 45 (p<0.001). Between treated groups at day 24, this level was decreased only in DE 200 compared to diabetic group (p<0.05) but at day 45 triglyceride levels of all extract-treated groups showed a significant decrease in comparison to the diabetic group (p<0.001 for all) (Table 2).

**Table 2:** Effect *of N. sativa *seed extract on average serum levels of cholesterol and triglyceride (mg/dl) in streptozotocin-induced diabetic rats

**Group**		**Cholesterol**						**Triglyceride**	
	**Day 0**	**Day 24**	**Day 45**	**Day 0**			**Day 24**		**Day 45**
**C**	71.91±2.83	73.66±1.21	72.63±4.17	55.42±2.58			87.38±4.31		56.43±2.44
**D**	76.05±1.29	108.62±1.7***	121.42±6.66***	61.91 ±6.40			293.39±7.82***		101.34±2.96***
**DM**	73±2.23	89.38±3.63###	82.27±3.23###	64.80±6.4			278.99±26.11***		267.60±21.31***
**DE 100**	70.07±3.79	87.43±4.22#	78.87±2.84###	65.03±5.56			248.56±13.15***		60.48±5.21###
**DE 200**	71.54±2.05	87.55±2.16###	86.25±3.22###	56.54±2.49			190.99±21.28*#		68.41±5.03###
**DE 400**	67.08±2.25	84.45±6.01###	80.60±5.60###	63.05±4.44			258.74±28.72***		65.82±17.28###

Abbreviations: C: control, D: diabetic, DM: diabetic + metformin, DE 100: diabetic + *N. sativa *extract of 100 mg/kg, DE 200: diabetic + *N. sativa *extract of 200 mg/kg, DE 400: diabetic + *N. sativa *extract of 400 mg/kg. Values are presented as means±SEM (n = 8).

∗ p<0.05,

*** p<0.001 compared to control group and

# p<0.05,

### p<0.001 compared to diabetic group.

**The contractile responses of aortic rings to phenylepherine**

**Figure 1:**
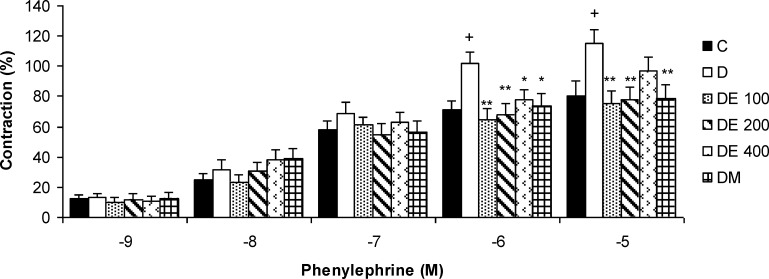
The contractile response of aortic rings to cumulative concentrations of phenylepherine (10^-9^ - 10^-5^ M) in control (C), diabetic (D), *N. sativa *seed extract (DE 100, DE 200, and DE 400), and metformin (DM) treated groups. Values are presented as means±SEM (n = 8). * p<0.05, ** p<0.01 compared to diabetic group and ^+ ^p<0.05 compared to control group

The contractile responses of aortic rings to cumulative concentrations of PE (10^-9^ to 10^-^^5^ M) were shown in Figure 1. The lower concentrations of *N. sativa* seed extract (DE 100 and DE 200) and metformin significantly reduced the contractile responses to higher concentrations of PE (10^-6 ^- 10^-5 ^M) compared to diabetic group (p<0.05 to p<0.01) (Figure 1).

**The contractile responses of aortic rings to KCl**

**Figure 2:**
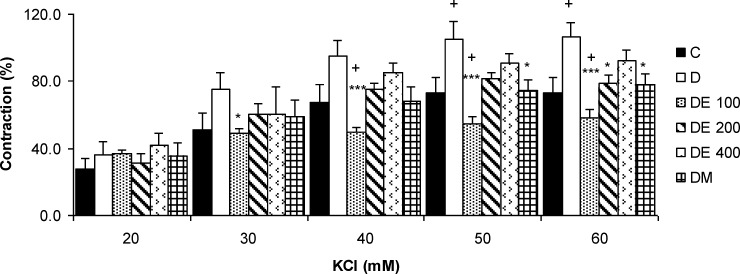
The contractile response of aortic rings to cumulative concentrations of KCL (20-60 mM) in control (C), diabetic (D), *N. sativa *seed extract (DE 100, DE 200, and DE 400), and metformin (DM) treated groups. Values are presented as means±SEM (n = 8). * p<0.05, *** p<0.001 compared to diabetic group and ^+^p<0.05 compared to control group

The cumulative concentrations of KCl (20-60 mM) increased the contractile responses of aortic rings concentration-dependently which were not significantly different between the groups (Figure 2).

**The relaxation responses of aortic rings to acetylcholine**

**Figure 3:**
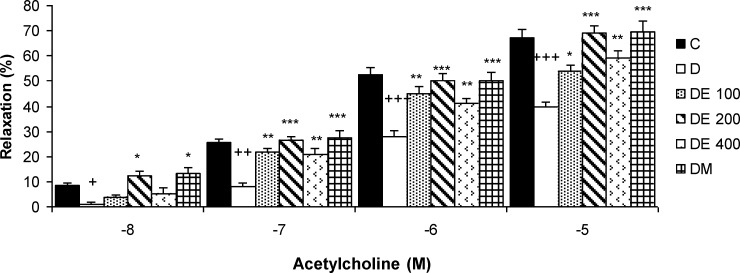
The relaxation responses of aortic rings to cumulative concentrations of acetylcholine (10^-8^ - 10^-5 ^ M) in control (C), diabetic (D), *N. sativa *seed extract (DE 100, DE 200, and DE 400), and metformin (DM) treated groups. Values are presented as means±SEM (n=8). *p<0.001, *** p<0.001 compared to diabetic group and ^+^ p<0.05, ^+++^ p<0.001 compared to control group

In aortic rings pre-contracted with PE, Ach relaxation was impaired in diabetic group compared to those from normal rats. The highest relaxation responses of aortic rings were observed in DE 200 and DM groups. The relaxation response to Ach 10^-8^ M, was increased in DE 200 and metformin groups compared to diabetic group (p<0.05). The relaxation responses to Ach 10^-7^** - **10^-5^ M, were significantly higher in all treated groups compared to diabetic group (p<0.05 to p<0.001) (Figure 3).

**The relaxation responses of aortic rings to** **sodium nitroprusside**

**Figure 4:**
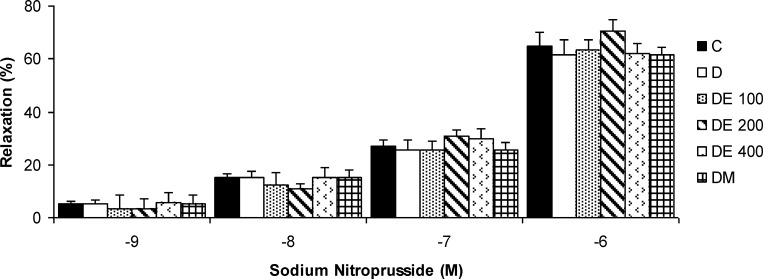
The relaxation responses of aortic rings to cumulative concentrations of sodium nitroprusside (10^-9^ - 10^-6^ M) in control (C), diabetic (D), *N. sativa *seed extract (DE 100, DE 200, and DE 400), and metformin (DM) treated groups. Values are presented as means±SEM (n = 8

In aortic rings pre-contracted with PE, the relaxation responses to SNP (10^-9^ - 10^-6^ M) were not significantly different between the groups (Figure 4).

## Discussion

The results of the present study indicate that chronic administration of *N. Sativa* seed extract has a significant hypoglycemic effect and improves aortic reactivity to vasoconstrictor and vasodilatation agents in STZ-induced diabetic rats. Previous studies demonstrated that enhanced vascular reactivity to vasoconstrictor agents (Pinho et al., 2010 ▶) or impairment of the vasodilation (Csanyi et al., 2007 ▶) contributes to the cardiovascular complications associated with DM. Promoted vascular reactivity to _1_ adrenoreceptor agonists was indicated in different vascular beds from diabetic animals (Abebe et al., 1990 ▶). The increased aortic contractile responses of diabetic rats may be due to impaired endothelial function (Potenza et al., 2009 ▶), increased calcium influx through voltage-dependent L-type Ca^2+^ channels (Pinho et al., 2010 ▶), increased myofilament Ca^2+^ sensitivity (Kizub et al., 2010 ▶), increased vasoconstrictor prostanoids due to increased superoxide anions, and increased sensitivity to adrenergic agonists (Ahmad and Beg, 2013 ▶) and oxidative stress (Tabit et al., 2010 ▶).

PE, an adrenoreceptor agonist, causes aortic contraction by Ca^2+^ influx via receptor-operated Ca2+ channels (ROCCs) and by release of Ca^2^^+^ from the sarcoplasmic reticulum (Thorneloe and Nelson, 2005 ▶; McCarron et al., 2003 ▶). The latter pathway involves PE stimulation of phospholipase C to produce diacylglycerol (DG) and 1,4,5 triphosphate inositol (IP_3_), and subsequently DG activates the light chain of myosin by activation of protein kinase C (PKC), and IP_3_ induces Ca^2+^ release from the sarcoplasmic reticulum through opening IP_3_ receptors (Thorneloe and Nelson, 2005 ▶). 

Results of previous studies have shown that the voltage-dependent Ca^2+^ channels (VDCCs) are involved in KCl-induced contraction. Our previous study showed the relaxant effect of *N. sativa* seed on the contractions induced by PE and KCl in VSMCs are mediated by inhibition of extracellular Ca^2+^ influx and also suppression of IP_3_-mediated receptors (Niazmand et al., 2014 ▶). Thus, the *N. sativa* effect on PE and KCl-induced vasoconstriction of diabetic rat aortic rings may be due to these effects. 

In endothelial cells of most vascular beds, Ach can stimulate formation and release of endothelial-derived relaxing factors including NO, prostacyclin, and endothelium-derived hyperpolarizing factor. This pathway causes the relaxation of vascular smooth muscle in an endothelium-dependent manner (Zhang et al., 2011 ▶). The Ach-induced relaxation response is endothelium-dependent and NO-mediated. The results of the present study revealed that the relaxant response was reduced in aortas from STZ-induced diabetic rats and this reduced relaxation was profoundly recovered by *N. sativa* seed treatment. The impairment of Ach-induced relaxation suggests a possible common pathophysiologic mechanism that there is an attenuation of NO release in the diabetic group, thus *N. sativa* seed may improve NO pathway in endothelial cells in diabetic rats.

Impaired endothelium-dependent relaxation in STZ-induced diabetic rat might be due to enhance blood glucose level and reduce blood insulin level. It has been shown that hyperglycemia leads to tissue damage by several mechanisms, including the advanced glycation end product (AGE) formation, increased polyol pathway flux, apoptosis, and reactive oxygen species (ROS) formation (Hartge et al., 2007 ▶). Our results showed that *N. sativa* treatment could exert a significantly hypoglycemic and hypolipidemic effects in STZ-induced diabetic rats. Therefore, its beneficial effect on aortic tissue of diabetic rats should be in part due to its hypoglycemic and hypolipidemic effects. Since the dosage of 200 mg/kg of *N. sativa* extract had the most beneficial effects on reducing blood glucose level, this hypoglycemic effect may be accompanied with decrease of AGE and explain the higher recovery of relaxant response in this case. Some damaging effects of diabetes on vascular tissue of diabetic animals are also believed to be due to promoted oxidative stress (Paneni et al., 2013 ▶). The initial trigger, i.e., high glucose concentrations change vascular function, is the imbalance between NO bioavailability and accumulation of ROS, which leads to endothelial dysfunction. Indeed, hyperglycemia-induced production of superoxide anion (O_2_^-^) inactivates NO to form peroxynitrite (ONOO^-^), a potent oxidant which easily penetrates across phospholipid membranes and induces substrate nitration (Luscher et al., 2003 ▶). The seed of *N. sativa* is well known for its powerful antioxidant properties (Ismail et al., 2010 ▶). Therefore, the other possible mechanism is the ability of *N. sativa* seed to reduce diabetic-induced oxidative stress damage on vascular tissues. 

In this study, we also evaluated the vascular effect of SNP, a general NO donor and an endothelium-independent vasodilator. The relaxation responses to SNP were similar and there was insignificant difference between our experimental groups. The majority of previous studies support our finding that the response of the tissue to SNP is not impaired in diabetics versus control (Elcioglu et al., 2010 ▶; Oyama et al., 1986 ▶).

Metformin is a classic drug for DM treatment. Previous studies have shown that metformin therapy was favorable for cardiovascular outcomes and the mechanisms might be partially related with its effects on improving insulin resistance, reducing serum level of C-reactive protein (CRP), enhancing eNOS expression and NO production, improving glycation and oxidative stress, and regulating glucose metabolism (Sena et al., 2011 ▶; Calvert et al., 2008 ▶; Liu et al., 2014 ▶). 

In the present study, although metformin treatment of STZ diabetic rats did not correct their increased blood glucose level, the altered responses to the vasoactive agents tested were corrected. Similar data were obtained in previous studies (Katakam et al., 2000 ▶; Majithiya and Balaraman, 2006 ▶; Sartoretto et al., 2005 ▶) that demonstrated improved vascular reactivity to endothelium-dependent vasodilator, acetylcholine, in diabetic rats after treatment with metformin. Metformin improved aortic reactivity of diabetic rats, which may be attributed to improved glycation and antioxidant defense and diminished Rho kinase activity. In diabetic preparations, involment of RhoA/Rho-kinase (ROCK) pathway in the mechanical activity of arteries via an increase in active RhoA-kinase level and decreases in both eNOS expression and NO production by endothelium has already been shown with the previously published data (Kizub et al., 2010 ▶; El-Saleh et al., 2004 ▶).

Our study demonstrated that chronic administration of *N. sativa* seed extract has a significant hypoglycemic effect and improved aortic reactivity to vasoconstrictor and vasodilator agents in STZ-induced diabetic rats.

## References

[B1] Abebe W, Harris KH, Macleod KM (1990). Enhanced contractile responses of arteries from diabetic rats to alpha 1-adrenoceptor stimulation in the absence and presence of extracellular calcium. J Cardiovasc Pharmacol.

[B2] Ahmad A, HusainA, Mujeeb M, Khan SA, Najmi AK, Siddique NA, Damanhouri ZA, Anwar F, Kishore K (2013). A review on therapeutic potential of Nigella sativa: A miracle herb. Asian Pac J Trop Biomed.

[B3] Ahmad S, Beg ZH (2013). Elucidation of mechanisms of actions of thymoquinone-enriched methanolic and volatile oil extracts from Nigella sativa against cardiovascular risk parameters in experimental hyperlipidemia. Lipids Health Dis.

[B4] Calvert JW, Gundewar S, Jha S, Greer JJ, Bestermann WH, Tian R, Lefer DJ (2008). Acute metformin therapy confers cardioprotection against myocardial infarction via AMPK-eNOS-mediated signaling. Diabetes.

[B5] Capellini VK, Baldo CF, Celotto AC, Batalhao ME, Carnio EC, Rodrigues AJ, Evora PR (2010). Oxidative stress is not associated with vascular dysfunction in a model of alloxan-induced diabetic rats. Arq Bras Endocrinol Metabol.

[B6] Csanyi G, Lepran I, Flesch T, Telegdy G, Szabo G, Mezei Z (2007). Lack of endothelium-derived hyperpolarizing factor (EDHF) up-regulation in endothelial dysfunction in aorta in diabetic rats. Pharmacol Rep.

[B7] De Vriese AS, Verbeuren TJ, Van De Voorde J, Lameire NH, Vanhoutte PM (2000). Endothelial dysfunction in diabetes. Br J Pharmacol.

[B8] Dehkordi FR, Kamkhah AF (2008). Antihypertensive effect of N. sativa seed extract in patients with mild hypertension. Fundam Clin Pharmacol.

[B9] El-Remessy AB, Tawfik HE, Matragoon S, Pillai B Caldwell RB, Caldwell RW (2010). Peroxynitrite mediates diabetes-induced endothelial dysfunction: possible role of Rho kinase activation. Exp Diabetes Res.

[B10] El-Saleh SC, Al-Sagair OA, Al-Khalaf MI (2004). Thymoquinone and Nigella sativa oil protection against methionine-induced hyperhomocysteinemia in rats. Int J Cardiol.

[B11] Elcioglu KH, Kabasakal L, Cetinel S, Conturk G, Sezen SF, Ayanoglu-Dulger G (2010). Changes in caveolin-1 expression and vasoreactivity in the aorta and corpus cavernosum of fructose and streptozotocin-induced diabetic rats. Eur J Pharmacol.

[B12] Fallah Huseini H, Amini M, Mohtashami R, Ghamarchehre ME, Sadeqhi Z, Kianbakht S, Fallah Huseini A (2013). Blood pressure lowering effect of Nigella sativa L. seed oil in healthy volunteers: a randomized, double-blind, placebo-controlled clinical trial. Phytother Res.

[B13] Ghosheh OA, Houdi AA, Crooks PA (1999). High performance liquid chromatographic analysis of the pharmacologically active quinones and related compounds in the oil of the black seed (Nigella sativa L.). J Pharm Biomed Anal.

[B14] Hartge MM, Unger T, Kintscher U (2007). The endothelium and vascular inflammation in diabetes. Diab Vasc Dis Res.

[B15] Ismail M, Al-Naqeep G, Chan KW (2010). Nigella sativa thymoquinone-rich fraction greatly improves plasma antioxidant capacity and expression of antioxidant genes in hypercholesterolemic rats. Free Radic Biol Med.

[B16] Kaleem M, Kirmani D, Asif M, Ahmed Q, Bano B (2006). Biochemical effects of Nigella sativa L seeds in diabetic rats. Indian J Exp Biol.

[B17] Katakam PV, Ujhelyi MR, Hoenig M, Miller AW (2000). Metformin improves vascular function in insulin-resistant rats. Hypertension.

[B18] Khazdair MR (2015). The Protective Effects of Nigella sativa and Its Constituents on Induced Neurotoxicity. J Toxicol.

[B19] Kizub IV, Pavlova OO, Johnson CD, Soloviev AI, Zholos AV (2010). Rho kinase and protein kinase C involvement in vascular smooth muscle myofilament calcium sensitization in arteries from diabetic rats. Br J Pharmacol.

[B20] Leong XF, Rais Mustafa M, Jaarin K (2013). Nigella sativa and Its Protective Role in Oxidative Stress and Hypertension. Evid Based Complement Alternat Med.

[B21] Liu Y, Huang C, Ceng C, Zhan H, Zheng D, Han W (2014). Metformin enhances nitric oxide production and diminishes Rho kinase activity in rats with hyperlipidemia. Lipids Health Dis.

[B22] Luscher TF, Creager MA, Beckman JA, Cosentino F (2003). Diabetes and vascular disease: pathophysiology, clinical consequences, and medical therapy: Part II. Circulation.

[B23] Majithiya JB, Balaraman R (2006). Metformin reduces blood pressure and restores endothelial function in aorta of streptozotocin-induced diabetic rats. Life sciences.

[B24] Mccarron JG, Bradley KN, Macmillan D, Muir TC (2003). Sarcolemma agonist-induced interactions between InsP3 and ryanodine receptors in Ca2+ oscillations and waves in smooth muscle. Biochem Soc Trans.

[B25] Nergiz C, Otles S (1993). Chemical composition of Nigella sativa L. seeds. Food Chemistry.

[B26] Niazmand S, Fereidouni E, Mahmoudabady M, Mousavi SM (2014). Endothelium-independent vasorelaxant effects of hydroalcoholic extract from Nigella sativa seed in rat aorta: the roles of Ca2+ and K+ channels. Biomed Res Int.

[B27] Oyama Y, Kawasaki H, Hattori Y, Kanno M (1986). Attenuation of endothelium-dependent relaxation in aorta from diabetic rats. Eur J Pharmacol.

[B28] Paneni F, Beckman JA, Creager MA, Cosentino F (2013). Diabetes and vascular disease: pathophysiology, clinical consequences, and medical therapy: part I. Eur Heart J.

[B29] Pinho JF, Medeiros, MA, Capettini LS, Rezende BA, Campos PP, Andrade SP, Cortes SF, Cruz JS, Lemos VS (2010). Phosphatidylinositol 3-kinase-delta up-regulates L-type Ca2+ currents and increases vascular contractility in a mouse model of type 1 diabetes. Br J Pharmacol.

[B30] Potenza MA, Gagliardi S, Nacci C, Carratu MR, Montagnani M (2009). Endothelial dysfunction in diabetes: from mechanisms to therapeutic targets. Curr Med Chem.

[B31] Sartoretto JL, Melo GA, Carvalho MH, Nigro D, Passaglia RT, Scavone C, Cuman RK, Fortes ZB (2005). Metformin treatment restores the altered microvascular reactivity in neonatal streptozotocin-induced diabetic rats increasing NOS activity, but not NOS expression. Life Sci.

[B32] Sena CM, Matafome P, Louro T, Nunes E, Fernandes R, Seica RM (2011). Metformin restores endothelial function in aorta of diabetic rats. Br J Pharmacol.

[B33] Tabit CE, Chung WB, Hamburg NM, Vita JA (2010). Endothelial dysfunction in diabetes mellitus: molecular mechanisms and clinical implications. Reviews in Endocrine and Metabolic Disorders.

[B34] Thorneloe KS, Nelson MT (2005). Ion channels in smooth muscle: regulators of intracellular calcium and contractility. Can J Physiol Pharmacol.

[B35] Xu J, Zou MH (2009). Molecular insights and therapeutic targets for diabetic endothelial dysfunction. Circulation Circulation.

[B36] Zhang LN, Vincelette J, Chen D, Gless RD, Anandan SK, Rubanyi GM, Webb HK, Macintyre DE, Wang YX (2011). Inhibition of soluble epoxide hydrolase attenuates endothelial dysfunction in animal models of diabetes, obesity and hypertension. Eur J Pharmacol.

[B37] Zou MH, Cohen R, Ullrich V (2004). Peroxynitrite and vascular endothelial dysfunction in diabetes mellitus. Endothelium.

